# Notes and News

**Published:** 1897-02-06

**Authors:** 


### NOTES AND NEWS.

(COLLECTED AND COMMUNICATED.)

H.R.H. THE PRINCESS OF WALES has consented to become patroness of the National Free Home for the Dying at Clapham, of which the Duchess of Sutherland is president, and the Bishop of Rochester patron. This home is absolutely free, and admits patients without distinction of creed or country.

THE new buildings which are being erected for the Guy's Hospital Medical School will be opened early in May by H.R.H. the Prince of Wales, the President of the hospital. It should be pointed out that these buildings have nothing to do with the hospital so far as the funds by which they are built is concerned, the money being provided by the school, which alone is responsible for the expense.

THE Hospital Saturday Fund has awarded to 33 general hospitals £6,661 5s., 64 special hospitals £6,218 14s., 37 dispensaries £982 19s., 20 convalescent homes £1,662 15s., 23 miscellaneous (including distribution and surgical appliance committees, also institutions for the gratuitous nursing of the sick poor in their own homes) £2,493 4s. The total receipts for the past year were £21,348, as against £20,039 during the previous twelve months. The awards were more than those of 1895 by £368.

THE Governors of the Coventry and Warwickshire Hospital have taken an obviously proper course in regard to the new rule, which provides that "no person shall be eligible as honorary medical officer unless he be registered under the Medical Act, and possesses both a medical and surgical qualification obtained from a University or other examining body in Great Britain or Ireland." At the meeting when the resolution was originally put a rider was added, which contained the stipulation that the rule should not come into effect until after the vacancy then existing on the medical staff should be filled. The Governors saw fit to rescind this rider at the last meeting of the board, and the rule will have immediate effect.

THE Lord Mayor will preside at a meeting in aid of Charing Cross Hospital, to be held at the Mansion House on Friday, March 5th, at three p.m.

THE Actors' Benevolent Fund has undertaken to receive the funds originally collected with a view to establishing a Home Hospital for the profession, and to carry out the intended scheme as far as possible.

MR. PASSMORE EDWARDS has offered £2,000 towards the erection of a cottage hospital at Acton to commemorate the Diamond Jubilee, and to be named after the Queen. The condition attached to the offer is that a site and endowment should be secured from other sources.

IN connection with the scheme to establish a new Consumption Hospital for Liverpool, it has been decided firstly to build a branch hospital, with twenty beds, in the country, and then to pull down the present hospital and build a new one to accommodate forty patients. About £50,000 will be required to complete the scheme, towards which £23,000 has been promised.

THE first number of the "Middlesex Hospital Journal," which has just been issued, gives promise of an interesting future. It contains an admirable portrait of the late Mr. Hulke, with a sketch of his career; two or three clinical lectures; a short history of the Middlesex Hospital Medical Society; and notes on many matters of interest to Middlesex students past and present.

IT seems almost incredible that a large city like Dublin should be so managed that when a single case of small-pox appears the whole machinery of the municipality is thrown into a state of confusion in an endeavour to find accommodation for the patient. Whilst the rusty municipal wheels were set going the patient, fortunately, arrived at convalescence in her own home, without so far spreading the contagion. The result of much serious deliberation between Guardians, Local Government Board, and Corporation is, that the Corporation is to be asked to conclude their purchase of the Pigeon House Fort, that the Guardians may take it off their hands as an infectious hospital—a proposal that was made long ago, it being then put forward that Dublin was in urgent need of an infectious hospital. For some time Dublin has slept happily on on the understanding that the Cork Street Hospital would take infectious cases sent by the Guardians; but, on this arrangement being tested in respect to the case in question, the result is, the Guardians are told that the hospital is full, and further inquiries showed that, had it not been so, a case of small-pox would not be admitted; and, further, that when small-pox was admitted to the hospital previously, it had proved a source of contagion to the neighbourhood. In spite of this last fact, the only course that suggests itself, in case of an epidemic, to the Guardians until they acquire Pigeon House Fort, is to supply the Cork Street Hospital with accommodation for a certain number of convalescents in exchange for accommodation for small-pox patients. Provision for the treatment of infectious disease is a difficult question everywhere, but there are not many places where the authorities have succeeded so effectually in ignoring it as at Dublin.

